# Beneficial Effects of Metformin and/or Salicylate on Palmitate- or TNFα-Induced Neuroinflammatory Marker and Neuropeptide Gene Regulation in Immortalized NPY/AgRP Neurons

**DOI:** 10.1371/journal.pone.0166973

**Published:** 2016-11-28

**Authors:** Wenqing Ye, Ernesto H. Ramos, Brian C. Wong, Denise D. Belsham

**Affiliations:** 1 Department of Physiology, Faculty of Medicine, University of Toronto, Toronto, Ontario, Canada; 2 Departments of Medicine and Obstetrics and Gynaecology, Faculty of Medicine, University of Toronto, Toronto, Ontario, Canada; Universidade de Santiago de Compostela, SPAIN

## Abstract

Neuropeptide Y (NPY)/Agouti-related peptide (AgRP)-expressing neurons in the hypothalamus induce feeding and decrease energy expenditure. With consumption of a diet high in fat, there is an increase in circulating saturated free fatty acids, including palmitate, leading to the development of neuroinflammation and secretion of cytokines, such as TNFα, and in turn activation of the canonical IKKβ/NFκB cascade. We describe a model of palmitate- and TNFα-induced neuroinflammation in a functionally characterized, immortalized NPY/AgRP-expressing cell model, mHypoE-46, to study whether the anti-diabetic metformin alone or in combination with the anti-inflammatory agent salicylate can ameliorate these detrimental effects. Treatment with palmitate increased mRNA expression of feeding peptides *Npy* and *Agrp*, and inflammatory cytokines *Tnfa* and *Il-6*, whereas treatment with TNFα increased mRNA expression of *Npy*, *Nfkb*, *Ikba*, *Tnfa*, and *Il-6*. The effects of metformin and/or sodium salicylate on these genes were assessed. Metformin increased phosphorylation of AMPK and S6K, while sodium salicylate increased phospho-AMPK and decreased phospho-S6K, but neither had any effect on phospho-ERK, -JNK or –p38 in the mHypoE-46 NPY/AgRP neurons. Furthermore, we utilized a pre-treatment and/or co-treatment paradigm to model potential clinical regimens. We determined co-treatment with metformin or sodium salicylate alone was successful in alleviating changes observed in feeding peptide mRNA regulation, whereas a preventative pre-treatment with metformin and sodium salicylate together was able to alleviate palmitate- and TNFα-induced induction of NPY and/or AgRP mRNA levels. These results highlight important differences in reactive versus preventative treatments on palmitate- and TNFα-induced neuroinflammation in NPY/AgRP neurons.

## Introduction

The hypothalamus plays a critical role in the regulation of feeding and energy homeostasis through the control of potent neuropeptides, especially neuropeptide Y, agouti-related peptide (NPY/AgRP) and proopiomelanocortin (POMC) [1]. In states of over-nutrition, there is rapid development of hypothalamic inflammation from the increased intake of saturated fatty acids (SFAs) [2, 3]. SFAs, such as the highly prevalent palmitate, act as ligands on toll-like receptors (TLRs) to activate the inhibitor of IkappaB kinase beta/nuclear factor kappa B (IKKβ/NFκB) signal transduction cascade [4–7], which leads to the increased expression of several pro-inflammatory cytokines, markedly tumor necrosis factor alpha (TNFα), interleukin (IL)-6, and IL-1β [3]. TNFα in particular has been demonstrated to be involved in the promotion of diet-induced metabolic syndromes through TNFα receptor (TNFR) pathway activation. For instance, direct intracerebroventricular injection of TNFα was shown repeatedly to increase food consumption, reduce energy expenditure, and impair pancreatic release of insulin [8–10]. Neuronal inflammation in the central nervous system (CNS) can disrupt energy regulation by impairing insulin sensitivity, secretion of neuropeptides, such as NPY and α-melanocortin-stimulating hormone, and perturb the glucose sensing abilities of POMC and NPY neurons [8, 11]. Furthermore, sustained elevated NPY/AgRP levels perpetuate a dysfunctional feeding circuit resulting in the development of diet-induced obesity (DIO) from overfeeding [2, 3, 12]. The genetic ablation of components within this canonical inflammatory pathway can lead to improvements in metabolic dysfunction, through a decrease in food intake and weight lost [13]. However, genetic ablation is not a practical therapy in clinics for patients who suffer from metabolic disorders, such as Type 2 Diabetes Mellitus (T2DM).

Clinically, metformin is given as the primary treatment for T2DM [14, 15]. In addition to metformin, patients are often encouraged to take a salicylate-based medication, such as low-dose aspirin or sodium salicylate in order to protect against macrovascular effects of T2DM [16]. *In vivo* studies investigating the mechanism behind the effect of metformin and sodium salicylate on NPY/AgRP neurons are difficult due to the heterogeneous cellular makeup of the hypothalamus, and have led to inconclusive results [17–21]. This necessitates *in vitro* hypothalamic cell models to determine the direct effects of metformin and salicylate on NPY/AgRP neurons, which our lab has generated and characterized [22, 23]. The goal of our study was to establish an *in vitro* model of neuroinflammation in NPY/AgRP expressing neurons, and to utilize a therapeutic approach in alleviating palmitate- and TNFα-induced inflammation. We found that palmitate and TNFα treatment resulted in an induction of orexigenic neuropeptide expression and other neuroinflammatory markers. The molecular pathway by which metformin or salicylate affect mHypoE-46 neurons appear to be mediated through 5’ adenosine monophosphate-activated protein kinase (AMPK) and P70-S6 kinase (S6K). Using either palmitate or TNFα, we pre- and/or co-treated with metformin, salicylate or the two drugs in combination. We demonstrate that the co-treatment results in a rescue of the palmitate- or TNFα-mediated increase in NPY and/or AgRP in mHypoE-46 NPY/AgRP neurons. Importantly, we found an added benefit of pre-treatment with metformin and salicylate over the co-treatment alone, particularly on *Npy* mRNA expression, indicating a protective effect of preventive treatment.

## Material and Methods

### Cell culture and reagents

Embryonic mouse hypothalamic neurons were immortalized as previously described [22, 23]. Mice (BALB/c females and DC1 males from Charles River Laboratories, Wilmington, MA) were bred and an entire litter was harvested at E17 for the mHypoE-46 line. The mothers were sacrificed by CO_2_ exposure and then the fetuses were immediately decapitated to remove the hypothalamii under a dissecting microscope. This was followed by culture of the primary neurons, immortalization of the neurons, and subcloning to single cell-derived lines. All experimental protocols were approved by the Animal Care Committee of the University Health Network, Toronto General Hospital. mHypoE-46 neurons were cultured in DMEM (Sigma-Aldrich, St. Louis, MO, USA) containing 1 mM glucose, supplemented with 5% fetal bovine serum (FBS), and 1% penicillin-streptomycin (Gibco, Burlington, ON, Canada). Cells were cultured in 5% CO_2_ at 37 C. Palmitate, TNFα, metformin and sodium salicylate were purchased from Sigma-Aldrich and diluted to their respective concentrations in molecular grade water (Thermo-Scientific, Nepean, ON, Canada). Salicylate was filter sterilized prior to treatment.

### Quantitative real-time RT-PCR (qRT-PCR)

mHypoE-46 neurons were grown to 70–80% confluency on 60 mm plates (Sarstedt, Montreal, QC, Canada) using 1 mM glucose supplemented with 5% FBS for 16 h experiments. For pre-treatments, mHypoE-46 cells were treated 1 h prior to palmitate or TNFα RNA was isolated using PureLink RNA Kit with on-column PureLink DNase (Ambion; Streetsville, Ontario, Canada). cDNA was then synthesized using the high capacity cDNA reverse transcription kit (Applied Biosystems, Life Technologies, Carlsbad, CA, USA). Amplification of 25 ng cDNA was then performed using qRT-PCR master mix (Platinum SYBR Green qPCR SuperMix-UDG with ROX; Applied Biosystems, Life Technologies) with gene specific primers. Samples were run as triplicates on Applied Biosystems Prism 7000 sequence Detection System. qRT-PCR data analysis was performed using standard curve method and normalized to the reference gene, histone 3A. Primer sequences are found in Table 1.

**Table 1:** Primer sequences used.

Gene name	Primer sequence (5’ -> 3’)	Amplicon Size (bp)	Annealing Temperature (°C)
*Histone (3a)*	F: CGC TTC CAG AGT GCA GCT AAT	72	60
	R: ATC TTC AAA AAG GCC AAC CAG AT		
*Npy*	F: CAG AAA ACG CCC CCA GAA	77	60
	R: AAA AGT CGG GAG AAC AAG TTT CAT T		
*Agrp*	F: CGG AGG TGC TAG ATC CAC AGA	69	60
	R: AGG ACT CGT GCA GCC TTA CAC		
*Il-6*	F: TCA CTG TGC GTT GCA AAC AGT GTC	103	60
	R: ATA CCA CAA GGT TGG CAG GTG GAT		
*Tnfα*	F: GCT GTA CCT TAT CTA CTC CC	103	60
	R: CTC CTG GTA TGA AAT GGC		
*Ikbα*	F: TGC CTG GCC AGT GTA GCA GTC TT	150	60
	R: CAA AGT CAC CAA GTG CTC CAC GAT		
*Nfkb*	F: GGA TGA CAG AGG CGT GTA TTA G	114	60
	R: CCT TCT CTC TGT CTG TGA GTTG G		

### Western blot analysis

Cells were grown to 70–80% confluency prior to treatment. Cells were incubated in 1% FBS overnight for 16 h and then exposed to metformin or salicylate. 1X cell lysis buffer (Cell Signaling Technology, Danvers, MA, USA), consisting of 1% protease inhibitor, 1% phosphatase inhibitor cocktail 2, and 1 mM PMSF (Sigma-Aldrich), was used to harvest protein. Harvested protein was then centrifuged at 14000x g for 10 min and the soluble fraction was isolated. Using the BCA assay (Thermo Fisher), protein concentration was measured. 15 μg of protein was run on a 10% SDS-PAGE gel and transferred on a 0.22 μM PVDF membrane (Bio-Rad, Missisuaga, ON, Canada). Subsequently, the membrane was blocked with 5% nonfat dry milk dissolved in Tris-buffered saline with 0.1% Tween 20 (TBS-T) for 1 h and incubated overnight at 4°C with 1:1000 dilutions of primary antibody. Next, membranes were washed in 0.1% TBS-T and incubated for 1 h in horseradish peroxidase-linked secondary anti-rabbit antibody (1:7500, CST) at room temperature. Visualization of western blots were accomplished using Signal Fire ECL reagent (Cell Signaling Technology) on a Kodak Image Station 2000R (Eastman Kodak Company, Rochester, NY, USA). Protein densitometry and analysis was performed on ImageJ (National Institute of Mental Health, Bethesda, MD, USA) using β-actin as loading control. Primary antibodies used include phospho-AMPK, AMPK, phospho-p44/42 (ERK1/2), p44/42 (ERK1/2), phospho-p70/S6K, p70/S6K, phospho-JNK, JNK, phospho-P38, P38 (CST) and β-actin (Sigma-Aldrich).

### Statistical analysis

Data was analyzed using GraphPad Prism 6.0 (GraphPad Software; La Jolla, CA) and shown as ± SEM. Experiments used either one- or two-way ANOVA, followed by Bonferroni’s *post-hoc* test. Significance is indicated with a p-value < 0.05.

## Results

### Palmitate induced *Npy*, *Agrp*, and inflammatory marker mRNA expression in mHypoE-46 neurons

**Fig 1:**
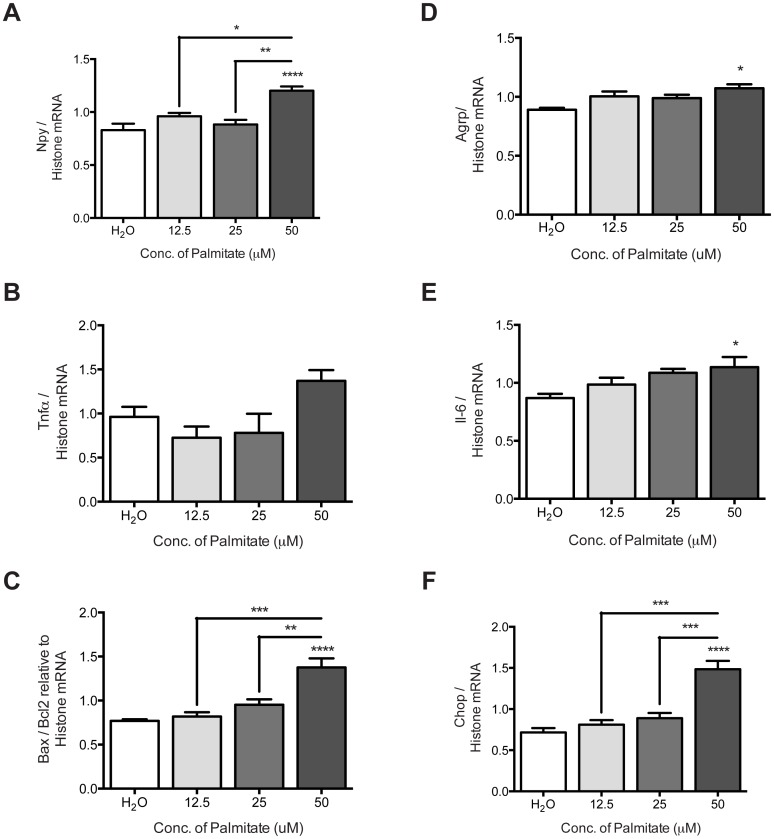
Transcription of feeding peptides and inflammation markers were regulated by treatment with palmitate in mHypoE-46 neurons. Cells were treated with 12.5, 25, 50 μM of palmitate or H_2_O vehicle for 16 h. RNA was isolated and DNase treated preceding cDNA generation for qRT-PCR. Relative mRNA expression was normalized to levels of histone 3A mRNA levels and expressed as mean ± SEM. 50 μM palmitate significantly increased *Npy* (A), *Bax/Bcl2* (C), *Agrp* (D), *Il-6* (E), and *Chop* (F) at 16h (*p<0.*05*, ****p<0.0001 by one-way ANOVA, n = 3–6 independent experiments) in comparison to H_2_O control. 50 μM palmitate significantly increased *Npy*, *Bax/Bcl2*, and *Chop* mRNA expression in comparison to 12.5 μM palmitate (*p<0.05, ***p<0.001 by one-way ANOVA, n = 3–6 independent experiments), and 25 μM palmitate (**p<0.01, ***p<0.001 by one-way ANOVA, n = 3–6 independent experiments). 50 μM of palmitate increased *Tnfα* mRNA expression, but this did not reach significance (p<0.4747 by one-way ANOVA, n = 3–6 independent experiments).

To determine the dose of palmitate that would elicit a regulatory effect on the mHypoE-46 neurons, we performed a dose curve with 12.5, 25, and 50 μM of palmitate and assessed mRNA transcript levels of feeding peptides *Npy* and *Agrp*, inflammatory markers *Tnfa* and *Il-6*, as well as cell death markers *Bax/Bcl2* and *Chop* at 16 h. Our results demonstrated that 50 μM of palmitate significantly up-regulated *Npy*, *Agrp*, *Il-6*, *Bax/Bcl2*, and *Chop* mRNA expression at 16 h in a dose-dependent manner (Fig 1A and 1C–1F). There was a trend to increasing *Tnfa* mRNA expression with palmitate treatment, but this did not reach significance (Fig 1B). Taken together, these results determined that the effective dose of palmitate that directly regulates transcriptional activity in the mHypoE-46 neurons is 50 μM.

### TNFα induced *Npy*, and inflammatory mRNA expression in mHypoE-46 neurons

**Fig 2:**
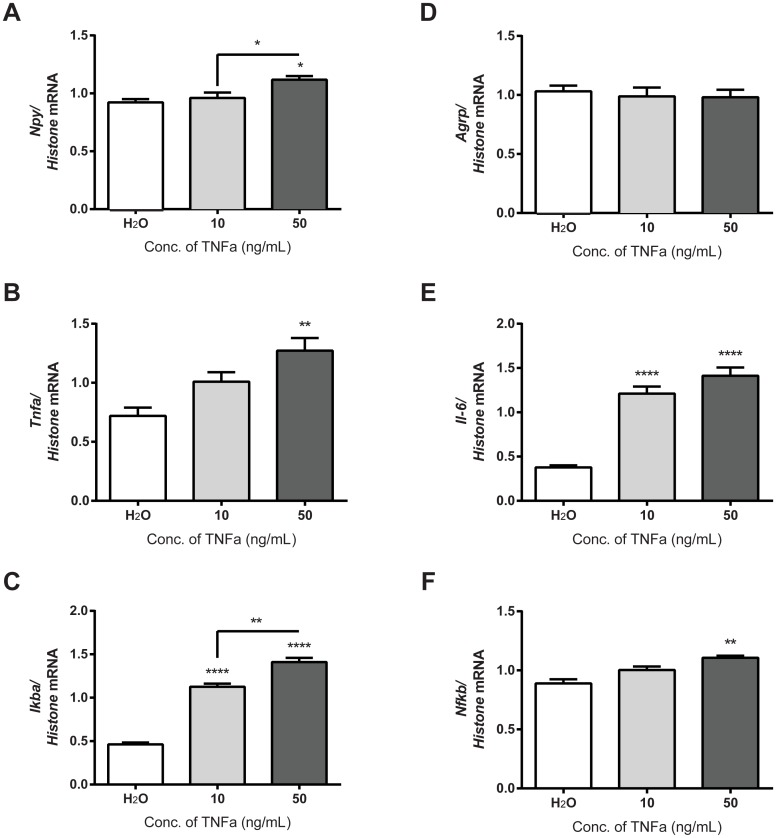
Transcription of feeding peptides and inflammation marks were regulated by treatment with TNFα in mHypoE-46 neurons. Cells were treated with 10, 50, ng/mL of TNFα or H_2_O vehicle for 6 h. RNA was isolated and DNase treated preceding cDNA generation for qRT-PCR. Relative mRNA expression was normalized to levels of histone 3A mRNA levels and expressed as mean ± SEM. 50 ng/mL TNFα significantly increased *Npy* (A), *Tnf*α (B), *I*κ*b*α (C), *Il-6* (E), and *Nf*κ*b* (F) mRNA expression at 6h (*p<0.*05*, **p<0.*01*, ****p<0.*0001* by one-way ANOVA, n = 3–4 independent experiments) in comparison to H_2_O control. 50 ng/mL TNFα significantly increased *Npy*, and *I*κ*b*α mRNA expression in comparison to 10 ng/mL TNFα (*p<0.*05*, **p<0.*01* by one-way ANOVA, n = 3–4 independent experiments). 10 ng/mL TNFα significantly increased *I*κ*b*α, and *Il-6* mRNA expression (****p<0.*0001* by one-way ANOVA, n = 3–4 independent experiments) in comparison to H_2_O control. TNFα did not regulate *Agrp* (D) mRNA expression at 6 h.

TNFα is a downstream protein product of the canonical inflammatory IKKβ/NFκB pathway activated by palmitate [24, 25]. We therefore studied the effects of TNFα treatment on the mHypoE-46 neurons. We performed a dose curve with 10 or 50 ng/mL of TNFα and studied the mRNA regulation of feeding peptides *Npy* and *Agrp*, as well as inflammatory markers *Tnfα*, *Il-6*, *Ikba*, and *Nfkb* at 6 h. We determined that 50 ng/mL of TNFα significantly up-regulated *Npy*, *Tnfa*, and *Nfkb* mRNA expression at 6 h (Fig 2A, 2B and 2F), with *Il-6* and *Ikba* mRNA expression exhibiting dose-dependency (Fig 2C and 2E). There was no significant difference in *Agrp* mRNA expression upon TNFα treatment. These results indicate the effective dose of TNFα that directly regulated mRNA expression in mHypoE-46 neurons is 50 ng/mL.

### Metformin and sodium salicylate regulated phosphorylation of AMPK and S6K in mHypoE-46 neurons

**Fig 3:**
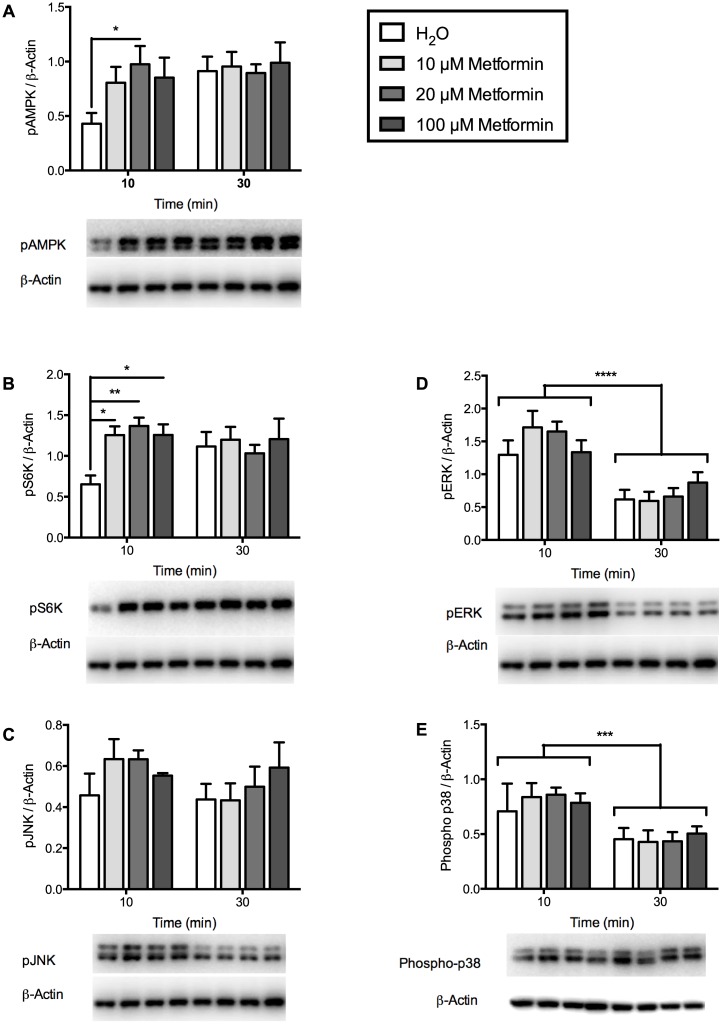
Phosphorylation of secondary-signaling molecules were regulated by metformin treatment in mHypoE-46 neurons. Cells were treated with 10, 20, 100 μM of metformin or H_2_O vehicle for 10 and 30 min. Protein was isolated for Western blot analysis. Levels of phospho-proteins were normalized to β-actin and expressed as mean ± SEM. Representative blots for phospho-proteins and β-actin are depicted beneath each graph. 20 μM metformin increased phosphorylation of AMPK (A) and S6K (B) at 10 min (*p<0.*05*, **p<0.*01* by two-way ANOVA, n = 4–6 independent experiments) in comparison to H_2_O control. Treatment with 10 or 100 μM metformin significantly increased S6K phosphorylation (*p<0.*05* by two-way ANOVA, n = 4–6 independent experiments) in comparison to H_2_O control. Phosphorylation of ERK (D) and p38 (E) was significantly decreased after 30 min (***p<0.*001*, ****p<0.*0001* by two-way ANOVA, n = 4–6 independent experiments). Metformin treatment did not regulate JNK phosphorylation (C).

**Fig 4:**
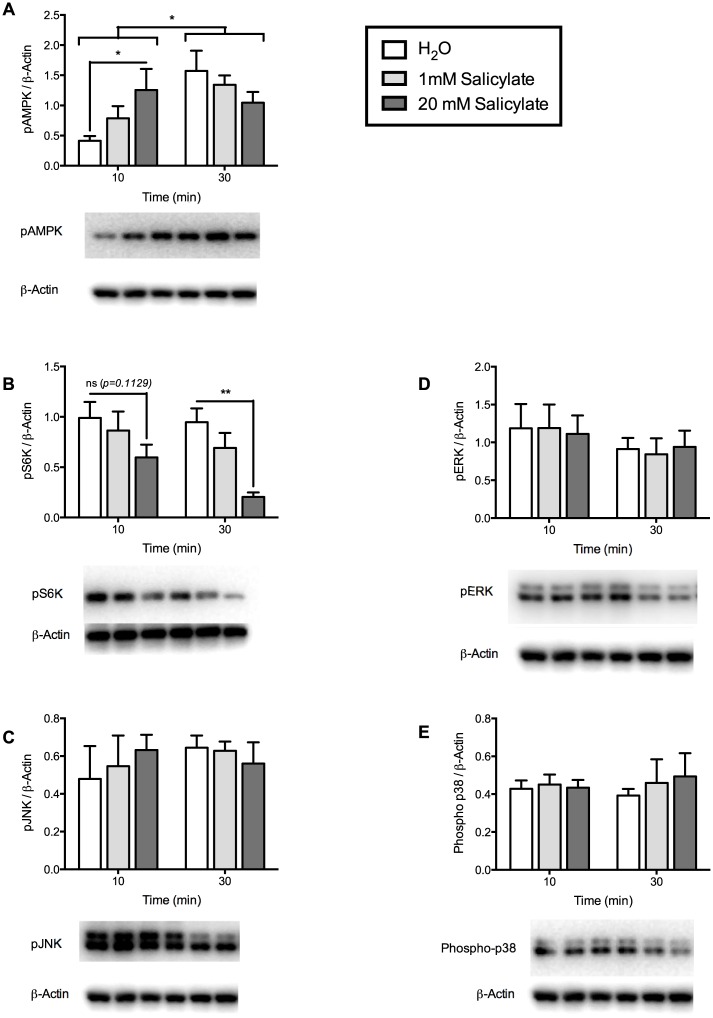
Phosphorylation of secondary-signaling molecules were regulated by sodium salicylate treatment in mHypoE-46 neurons. Cells were treated with 1 or 20 mM of sodium salicylate or H_2_O vehicle for 10 and 30 min. Protein was isolated for Western blot analysis. Levels of phospho-proteins were normalized to β-actin and expressed as mean ± SEM. Representative blots for phospho-proteins and β-actin are depicted beneath each graph. 20 mM sodium salicylate increased AMPK (A) phosphorylation and decreased S6K (B) phosphorylation at 10 (*p<0.*05* by two-way ANOVA, n = 4–6 independent experiments) and 30 min (**p<0.*01* by two-way ANOVA, n = 4–6 independent experiments) respectively. Sodium salicylate did not regulate phosphorylation of JNK, ERK, or p38 protein (C—E).

Given the ability of palmitate and TNFα treatment to induce dysregulation of important feeding neuropeptides and pro-inflammatory mRNA expression in the mHypoE-46 neurons, we assessed the ability of anti-diabetic drug metformin, and anti-inflammatory drug sodium salicylate to modulate the changes observed in mRNA expression. To determine the effective concentration of metformin and sodium salicylate, the mHypoE-46 neurons were treated with 10, 20 or 100 μM of metformin and 1 or 20 mM of sodium salicylate, and protein was isolated at 10 and 30 min. Metformin significantly increased AMPK phosphorylation at 20 μM, and S6K phosphorylation at all concentrations at 10 min (Fig 3A and 3B). Treatment with sodium salicylate significantly increased AMPK phosphorylation at 10 min in a dose-dependent manner (Fig 4A). Additionally, sodium salicylate decreased phosphorylation of AMPK at 30 min, and S6K at 10 and 30 min in a dose-dependent manner (Fig 4A and 4B). Treatment with metformin or sodium salicylate did not have any effects on the phosphorylation of ERK1/2, JNK, or p38 protein (Figs 3C–3E and 4C–4E). These results indicate that the effective doses of metformin and sodium salicylate in the mHypoE-46 neurons were 20 μM and 1 mM, respectively.

### Effects of metformin and sodium salicylate pre- and/or co-treatment on palmitate-induced mRNA regulation in mHypoE-46 neurons

**Fig 5:**
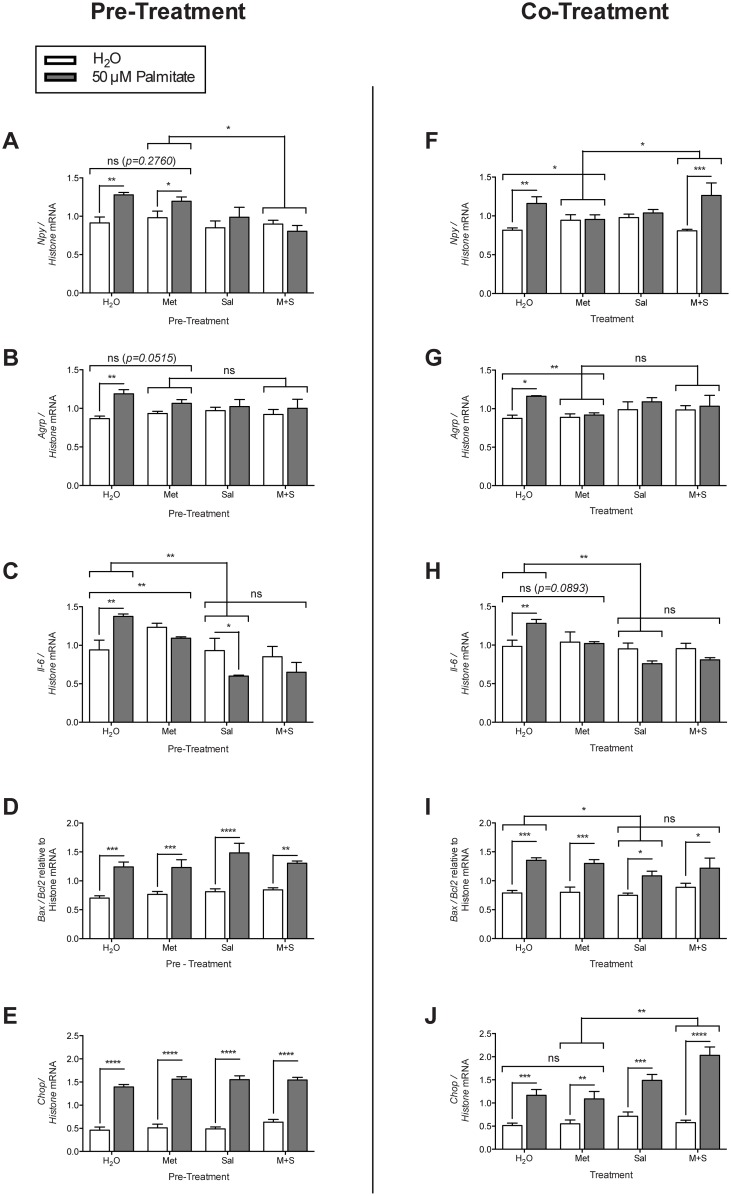
Effects of metformin and/or sodium salicylate on palmitate-induced inflammation in mHypoE-46 neurons. Cells were either (1.) pre-treated with 20 mM of sodium salicylate and/or 20 μM of metformin for 1 h followed 50 μM of palmitate or H_2_O vehicle for 16 h or (2.) co-treated with 20 mM of sodium salicylate and/or 20 μM of metformin with 50 μM of palmitate or H_2_O vehicle simultaneously. RNA was isolated and DNase treated preceding cDNA generation for qRT-PCR. Relative mRNA expression was normalized to levels of histone 3A mRNA levels and expressed as mean ± SEM. Pre-treatment with metformin alone attenuated the palmitate-induced upregulation in *Agrp* (B) and *Il-6* (C) mRNA levels (*p = 0*.*0515* and **p<0.*01* respectively by two-way ANOVA, n = 3–4 independent experiments). Pre-treatment with sodium salicylate alone attenuated the palmitate-induced up-regulation in *Npy* (A), *Agrp* (B), and *Il-6* (C) mRNA levels (*p<0.*05* by two-way ANOVA, n = 3–4 independent experiments). A combined pre-treatment with metformin and sodium salicylate attenuated the palmitate-induced increase in *Npy* (A), *Agrp* (B), and *Il-6* (C) mRNA levels. There was no significant change in *Bax/Bcl2* (D) and *Chop* (E) mRNA levels with sodium salicylate and/or metformin pre-treatment. Co-treatment with metformin alone attenuated the palmitate-induced upregulation of *Npy* (F), *Agrp* (G), and *Il-6* (H) mRNA (*p<0.*05*, **p<0.*01*, *p = 0*.*0893* respectively by two-way ANOVA, n = 3–4 independent experiments). Co-treatment with sodium salicylate alone attenuated the palmitate-induced increase in *Npy* (F), *Agrp* (G), *Il-6* (H), and *Bax/Bcl2* (I) (*p<0.*05*, **p<0.*01* by two-way ANOVA, n = 3–4 independent experiments). Co-treatment with metformin and sodium salicylate increased the palmitate-induced upregulated of *Npy* (F) mRNA (*p<0.05 by two-way ANOVA, n = 3–4 independent experiments), and attenuated the palmitate-induced upregulation of *Agrp* (G), *Il-6* (H), and *Bax/Bcl2* (I) mRNA. The palmitate-induced increase in *Chop* (J) mRNA levels were further increased with sodium salicylate and metformin co-treatment.

To assess the ability of metformin and sodium salicylate to mediate palmitate-induced transcriptional effects in the mHypoE-46 neurons, we used two different experimental paradigms. The mHypoE-46 neurons were either (1.) co-treated with 20 μM of metformin and/or 1 mM sodium salicylate and 50 μM palmitate for 16 h; or (2.) pre-treated for 1 h with 20 μM of metformin and/or 1 mM sodium salicylate followed by co-treatment with each drug and 50 μM palmitate for 16 h. Metformin pre-treatment significantly abolished the palmitate-induced up-regulation in *Agrp* and *Il-6* mRNA expression (Fig 5B and 5C); whereas co-treatment with metformin significantly blocked the up-regulation of *Npy*, *Agrp*, and *Il-6* mRNA levels (Fig 5F–5H). Sodium salicylate pre-treatment significantly abolished the palmitate-mediated up-regulation of *Npy* and *Agrp* mRNA expression, and significantly down-regulated *Il-6* mRNA expression (Fig 5A–5C). Sodium salicylate co-treatment significantly attenuated the palmitate-induced up-regulation of *Npy* and *Agrp* mRNA expression (Fig 5F and 5G), and significantly reduced *Il-6* and *Bax/Bcl2* mRNA expression (Fig 5H and 5I). A combined pre-treatment of metformin and sodium salicylate significantly blocked the palmitate-induced up-regulation of *Npy*, *Agrp*, and *Il-6* mRNA expression (Fig 5A–5C). Co-treatment with metformin and sodium salicylate significantly abolished palmitate-mediated up-regulation of *Agrp* and *Il-6* mRNA levels (Fig 5G and 5H). The palmitate-induced increase in *Chop* mRNA levels were further increased with sodium salicylate and metformin co-treatment (Fig 5J). Overall, the results illustrate the differential effects of metformin and/or sodium salicylate treatment, as well as the differences between pre-treatment versus co-treatment paradigms with the major difference being the significant block of the palmitate-mediated increase in NPY only in the pre-treatment paradigm with combination of drugs.

### Effects of metformin and sodium salicylate pre- and co-treatment on TNFα-induced mRNA regulation in mHypoE-46 neurons

**Fig 6:**
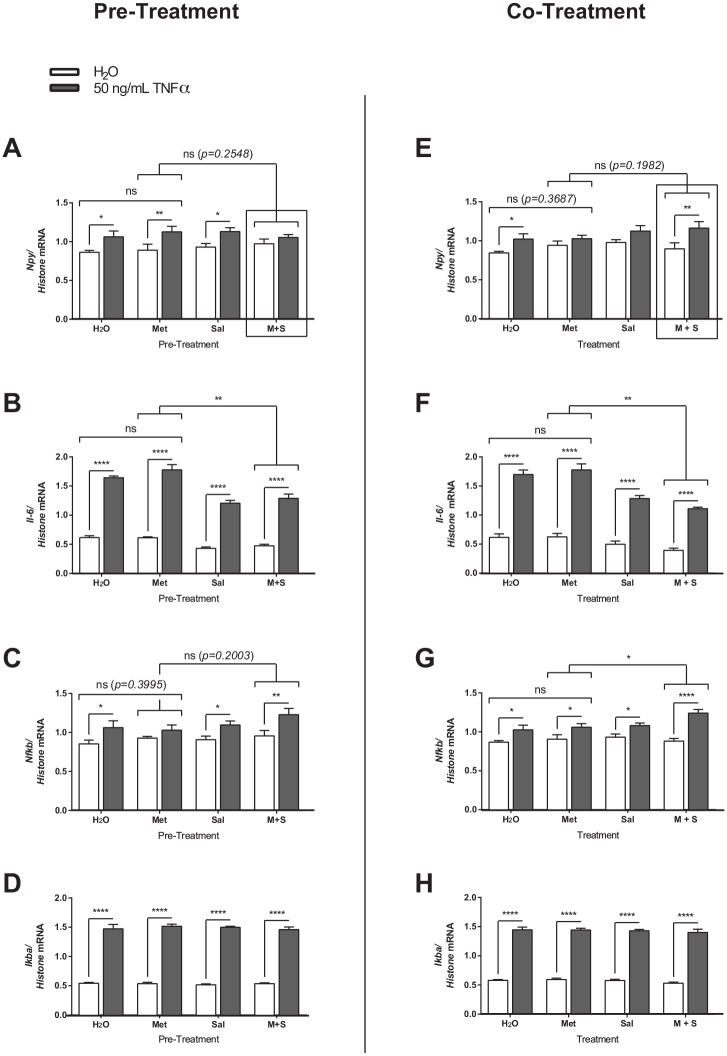
Effects of metformin and/or sodium salicylate on TNFα-induced inflammation in mHypoE-46 neurons. Cells were either (1.) pre-treated with 20 mM of sodium salicylate and/or 20 μM of metformin for 1 h followed 50 ng/mL of TNFα or H_2_O vehicle for 6 h or (2.) co-treated with 20 mM of sodium salicylate and/or 20 μM of metformin with 50 ng/mL of TNFα or H_2_O vehicle simultaneously. RNA was isolated and DNase treated preceding cDNA generation for qRT-PCR. Relative mRNA expression was normalized to levels of histone 3A mRNA levels and expressed as mean ± SEM. Pre-treatment with metformin alone attenuated the TNFα-induced upregulation in *Nfkb* (C) mRNA levels (*p = 0*.*3995* by two-way ANOVA, n = 4–6 independent experiments). Pre-treatment with sodium salicylate alone attenuated the TNFα-induced up-regulation in *Il-6* (B) mRNA levels (**p<0.*01* by two-way ANOVA, n = 3–4 independent experiments). A combined pre-treatment with metformin and sodium salicylate attenuated the TNFα-induced increase in *Npy* (A), and *Il-6* (B) mRNA levels, and upregulated the increase in *Nfkb* (C) mRNA levels. Co-treatment with metformin alone attenuated the TNFα-induced upregulation of *Npy* (F) mRNA (*p = 0*.*3687* respectively by two-way ANOVA, n = 4–6 independent experiments). Co-treatment with sodium salicylate alone attenuated the TNFα-induced increase in *Il-6* (F) mRNA levels (**p<0.*01* by two-way ANOVA, n = 4–6 independent experiments). Co-treatment with metformin and sodium salicylate increased the TNFα-induced upregulated of *Npy* (E) and *Nfkb* (G) mRNA (*p = 0*.*1982*, and *p<0.*05* respectively by two-way ANOVA, n = 4–6 independent experiments), and attenuated the TNFα-induced upregulation of *Il-6* (F) mRNA levels (**p<0.*01* by two-way ANOVA, n = 4–6 independent experiments). There was no significant change in *IkBa* (D, H) mRNA levels with sodium salicylate and/or metformin pre- and co-treatment.

**Fig 7:**
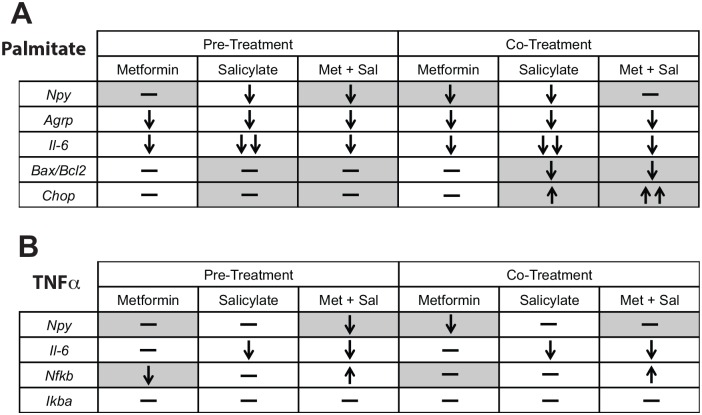
Summary of the effects of metformin and/or salicylate on the palmitate- or TNFα-mediated neuroinflammatory response and neuropeptide regulation in mHypoE-46 neurons. The results of pre-treatment and/or co-treatment with metformin and/or salicylate on (A) palmitate-mediated and (B) TNFα- induced changes in cytokine (*Il-6*, *Nfkb*, *IkBa*), ER stress marker (*Bax/Bcl*, *Chop*), and neuropeptide (*Npy*, *Agrp*) gene expression are shown in the Tables. The grey boxes signify differential effects on specific genes between the pre-treatment versus co-treatment paradigms.

To study the effects of metformin and sodium salicylate action on TNFα-mediated mRNA regulation, the mHypoE-46 neurons were exposed to a similar paradigm as described above. The mHypoE-46 neurons were either (1.) co-treated with 20 μM of metformin and/or 1 mM sodium salicylate and 50 ng/mL TNFα for 6 h; or (2.) pre-treated for 1 h with 20 μM of metformin and/or 1 mM sodium salicylate followed by co-treatment with each drug and 50 ng/mL TNFα for 6 h. Metformin pre-treatment significantly attenuated the TNFα-mediated up-regulation in *Nfkb* mRNA expression (Fig 6C); whereas co-treatment with metformin abolished the TNFα-mediated up-regulation in *Npy* mRNA levels (Fig 6E). Sodium salicylate pre-treatment and co-treatment significantly decreased the TNFα-mediated up-regulation of *Il-6* mRNA expression (Fig 6B–6F). Further, co-treatment with sodium salicylate also significantly blocked the TNFα-mediated up-regulation of *Npy* mRNA (Fig 6E). A pre-treatment with a combination of metformin and sodium salicylate significantly attenuated the TNFα-mediated up-regulation of *Npy* and *Il-6* mRNA expression (Fig 6A and 6B). On the other hand, co-treatment with metformin and sodium salicylate combined had no effect on the TNFα-mediated up-regulation in *Npy* or *Nfkb* mRNA expression (Fig 6E–6G), and significantly attenuated *Il-6* mRNA expression (Fig 6F). There were no changes in *Ikba* mRNA expression with pre-treatment or co-treatment paradigms (Fig 6D–6H). A summary and comparison of all of the findings is depicted in Fig 7. Again, the major difference between the pre- and co-treatments being the significant block of the TNFα-mediated increase in NPY by a combination of metformin and salicylate only in the pre-treatment paradigm. Interestingly, metformin alone was able to block the TNFα-mediated increase in *Npy* only in the co-treatment paradigm, indicating that there are specific differences between a pre- and co-treatment regimen.

## Discussion

Disruption of energy homeostasis by increased caloric intake and altered physiological responses to metabolic hormones at the level of the hypothalamus play a major role in the pathogenesis of T2DM and DIO [26, 27]. We therefore studied the effects of the anti-diabetic drug metformin and the anti-inflammatory drug sodium salicylate on diet-induced neuroinflammation at the level of the NPY/AgRP neuron in the hypothalamus. The mHypoE-46 cell-line was chosen as an appropriate model of NPY/AgRP expressing neurons due to previous work characterizing their expression of NPY, AgRP, as well as their physiological response to insulin, leptin, and other metabolic hormones [28–30]. We have demonstrated an increase in the mRNA expression of feeding peptides *Npy* and *Agrp* upon treatment with palmitate. This is consistent with previous *in vivo* studies looking at the expression of *Npy* in diet-induced obese mice [31, 32], as well as *in vitro* studies looking at the effects of palmitate on *Npy* regulation [28–30]. Dysregulation of *Npy* and *Agrp* mRNA expression can lead to an increase in feeding and obesity. Hypothalamic inflammation also contributes to the early development of T2DM through the dysregulation of insulin signaling in the hypothalamus [8, 10, 13]. Indeed, impairment of insulin signaling in hypothalamic neurons has also been shown at the cellular level [30]. Following central insulin signaling impairment, *in* vivo studies demonstrate a reduction in pancreatic insulin secretion culminating with T2DM [9]. Additionally, treatment with palmitate at high doses elicited a lipotoxic effect characterized by increased mRNA expression of cytokines *Tnfa* and *Il-6*, endoplasmic reticulum (ER) stress marker *Chop*, and apoptosis marker *Bax/Bcl2* [33]. *Tnfa* and *Il-6* mRNA expression has been implicated in the development of inflammatory-mediated DIO [34]. ER stress and activation of the canonical inflammatory cascade has been linked to the development of energy imbalance and DIO [35]. Prolonged and unresolved ER stress elicited by palmitate can also lead to impaired central response to feeding hormones, such as leptin [36]. Additionally, increased misfolding of proteins induced by ER stress can disrupt regular cell function and regulation [37, 38].

Due to the increased *Tnfa* mRNA expression observed with palmitate treatment in the mHypoE-46 neurons, we also studied the effects of TNFα-induced inflammation. We performed a dose curve using two concentrations of TNFα that appropriately model the low chronic inflammation that may be observed in individuals suffering from DIO [39, 40]. We have shown an increase in inflammatory markers *Tnfa*, *Il-6*, *Ikba*, and *Nfkb* upon TNFα treatment; thus demonstrating TNFα can be used as a surrogate to induce neuroinflammation. TNFα mediates signaling through the TNFR by activating the canonical IKKβ/NFκB pathway, leading to increased synthesis of *Tnfa* mRNA. Whereas NFκB activity leads to transcription of *Ikba*, which mediates intracellular inflammatory pathways [41, 42]. TNFα increases mRNA expression of *Npy*, but not *Agrp*. NPY and AgRP are co-expressed in the hypothalamus and play similar roles to increase food intake and decrease energy expenditure. AgRP is a strong appetite stimulator, while NPY acts to increase food intake and to increase fat storage [43]. Although the regulation of these feeding peptides are closely linked, it has been shown that NPY and AgRP can be differentially regulated under some circumstances [44]. Inactivation of *Tnfa* alone is not sufficient to protect against dysregulation of insulin signaling observed in models of DIO [45]. The difference in mRNA regulation of TNFα and palmitate suggest two pathways that may be simultaneous activated to mediate physiological changes in energy homeostasis and feeding pathways.

We have demonstrated that treatment with 20 μM of metformin increases AMPK and S6K phosphorylation confirming that metformin is able to regulate signaling pathways within NPY/AgRP-expressing neurons. In the periphery, the primary mechanism of action of metformin is mediated by AMPK to suppress gluconeogenesis in the liver, increase insulin sensitivity, and enhance glucose uptake [46, 47]. Metformin has also been shown to have direct anti-inflammatory properties, besides those related to its role in increasing insulin sensitivity [48]. However, the role of metformin in the central nervous system and how it is transported into neurons, specifically in the hypothalamus, is unknown. Activation of AMPK in NPY/AgRP neurons has demonstrated a beneficial role in attenuating elevated NPY levels [28], and plays a preventative role in the induction of insulin resistance with palmitate treatment [49]. We did not observe any changes to *Npy* and *Agrp* mRNA levels with metformin treatment alone, suggesting the activation of AMPK with metformin observed in the mHypoE-46 neurons may not be sufficient to regulate changes in these feeding peptides. Supplementary to metformin therapy, T2DM patients often take low dose salicylate-based drugs to reduce the risk of cardiovascular disease, since these have anti-inflammatory actions [16]. We have established sodium salicylate can regulate intracellular signaling cascades in NPY/AgRP expressing mHypoE-46 neurons by increasing phosphorylation of AMPK and decreasing phosphorylation of S6K. It has been shown that high doses of sodium salicylate can have positive effects on glucose regulation, and insulin sensitivity in T2DM [50–52]; however, there is a strong level of toxicity involved with high levels of sodium salicylate intake [53] and the specific effects of sodium salicylate on NPY/AgRP neurons in the hypothalamus has not been studied. Overall, we have shown both metformin and sodium salicylate can elicit cellular activity in NPY/AgRP expressing mHypoE-46 neurons by activation of downstream signaling pathways.

Due to the combination of metformin and sodium salicylate used clinically, we became interested in the effects this combination of drugs had on the NPY/AgRP expressing neurons of the hypothalamus. To appropriately model the clinical scenario *in vitro*, we studied two experimental paradigms (1.) A co-treatment of the drugs simultaneously with palmitate or TNFα was used to model current clinical models concerning alleviation of inflammatory pathways, and (2.) a pre-treatment model that utilizes the drug cocktail as a preventative measure to inhibit the activation of palmitate- or TNFα-mediated inflammatory pathways. We observed differences between the two paradigms in their ability to alleviate palmitate- and TNFα-induced inflammation. Co-treatment of metformin or sodium salicylate alone was successful in alleviating the palmitate- and TNFα-mediated dysregulation of *Npy* and *Agrp*; interestingly, a combination of metformin and sodium salicylate exacerbated the dysregulation of these feeding peptides in the co-treatment paradigm. Furthermore, results from the pre-treatment paradigm demonstrated an opposite effect on palmitate- and TNFα-induced neuroinflammation. Palmitate- and TNFα-mediated induction of *Npy* and *Agrp* mRNA was significantly decreased with metformin and sodium salicylate pre-treatment. However, pre-treatment with metformin or sodium salicylate alone had no effect on neuropeptide mRNA regulation.

It has been reported that salicylate activates AMPK, and may therefore mediate its beneficial effects on inflammation and metabolism through AMPK [54, 55]. In a recent study by Ford *et al*, the combinatory effects of metformin and salicylate were demonstrated to produce a synergistic effect in primary human hepatocytes *in vivo* and in clinical trials by improving insulin sensitivity, and reducing liver lipogenesis through AMPK [56]. Although this study provides the first insight into the synergistic effects of metformin and salicylate peripherally, it does not address the potential effects of the combined therapy in the brain despite the fact that both metformin and salicylate are able to cross the blood brain barrier [57, 58]. Overall, we have demonstrated an important difference in reactive versus preventative options in regards to alleviating neuroinflammation in NPY/AgRP expressing mHypoE-46 neurons.

The neuroinflammatory effects of palmitate and TNFα have not been studied in the hypothalamus, and the mechanisms involved are not yet defined. Whether well known anti-diabetic or anti-inflammatory compounds can act at the level of the hypothalamus to alleviate neuroinflammation and neuropeptide dysregulation caused by exposure to high levels of dietary fat is unclear. We have thus used a model of NPY/AgRP neurons to understand how these compounds act on individual neurons. In addition to NPY/AgRP neurons, POMC/CART-expressing neurons in the hypothalamus play an equally important role in feeding regulation and energy homeostasis. Neuroinflammation has been shown to dysregulate vital post-translational processing of POMC, leading to downstream abnormalities in feeding behaviour [59]. It would be valuable to further characterize the response of POMC/CART neurons to palmitate and TNFα to gain insight into the effect of metformin and sodium salicylate on this cell population. Overall, these therapeutics may have the potential to prevent the detrimental onset of T2DM and obesity if taken as a preventive measure in populations with a propensity towards insulin resistance and metabolic disorders.
